# Targeting the Highly Expressed microRNA miR-146b with CRISPR/Cas9n Gene Editing System in Thyroid Cancer

**DOI:** 10.3390/ijms22157992

**Published:** 2021-07-27

**Authors:** Daniel Casartelli de Santa-Inez, Cesar Seigi Fuziwara, Kelly Cristina Saito, Edna Teruko Kimura

**Affiliations:** Department of Cell and Developmental Biology, Institute of Biomedical Sciences, University of Sao Paulo, Sao Paulo 05508-000, Brazil; danielcsi@icb.usp.br (D.C.d.S.-I.); saito@icb.usp.br (K.C.S.)

**Keywords:** CRISPR/Cas9n, *miR-146b*, anaplastic thyroid cancer, microRNA, gene editing

## Abstract

Thyroid cancer is the most common endocrine malignancy, and the characterization of the genetic alterations in coding-genes that drive thyroid cancer are well consolidated in MAPK signaling. In the context of non-coding RNAs, microRNAs (miRNAs) are small non-coding RNAs that, when deregulated, cooperate to promote tumorigenesis by targeting mRNAs, many of which are proto-oncogenes and tumor suppressors. In thyroid cancer, *miR-146b-5p* is the most overexpressed miRNA associated with tumor aggressiveness and progression, while the antisense blocking of *miR-146b-5p* results in anti-tumoral effect. Therefore, inactivating *miR-146b* has been considered as a promising strategy in thyroid cancer therapy. Here, we applied the CRISPR/Cas9n editing system to target the *MIR146B* gene in an aggressive anaplastic thyroid cancer (ATC) cell line. For that, we designed two single-guide RNAs cloned into plasmids to direct Cas9 nickase (Cas9n) to the genomic region of the *pre-mir-146b* structure to target *miR-146b*-*5p* and *miR-146b*-*3p* sequences. In this plasmidial strategy, we cotransfected pSp-Cas9n-*miR-146b*-GuideA-puromycin and pSp-Cas9n-*miR-146b*-GuideB-GFP plasmids in KTC2 cells and selected the puromycin resistant + GFP positive clones (KTC2-Cl). As a result, we observed that the ATC cell line KTC2-Cl1 showed a 60% decrease in the expression of *miR-146b-5p* compared to the control, also showing reduced cell viability, migration, colony formation, and blockage of tumor development in immunocompromised mice. The analysis of the *MIR146B* edited sequence shows a 5 nt deletion in the *miR-146b-5p* region and a 1 nt deletion in the *miR-146b-3p* region in KTC2-Cl1. Thus, we developed an effective CRISPR/Cas9n system to edit the *MIR146B* miRNA gene and reduce *miR-146b-5p* expression which constitutes a potential molecular tool for the investigation of miRNAs function in thyroid cancer.

## 1. Introduction

The CRISPR (clustered regularly interspaced short palindromic repeats)/Cas9 (CRISPR Associated Protein 9) is a revolutionary methodology to modify genes using a single-guide RNA (sgRNA) and the endonuclease Cas9 [1]. In this system, Cas9 generates a double-strand break (DSB) in DNA guided by the sgRNA pairing to genomic regions adjacent to PAM sequences (NGG, for Sp.Cas9). The DNA repair system promptly fixes the DSB predominantly using the non-homologous end joining (NHEJ) pathway which is error prone (deletions, insertions, etc.) and may lead to gene truncation/inactivation [2]. This outcome is, therefore, the main use of CRISPR/Cas9 for depleting gene function which is essential for the studies of gene function, particularly in cancer.

Thyroid cancer is the most common endocrine malignancy [3], with the majority of cases comprising of the papillary thyroid cancer (PTC) histotype, with a good prognosis; and, on the other side, the rare anaplastic thyroid cancer (ATC), which is the most aggressive and lethal histotype. The characterization of the genetic alterations that drive thyroid cancer are well consolidated in MAPK signaling, and the BRAF^V600E^ mutation is the most frequent genetic event in PTC [4,5,6].

The advent of CRISPR/Cas9-mediated gene editing is a promising tool to understand thyroid cancer; however, this is a nascent field in thyroidology. In the context of non-coding RNAs, microRNAs (miRNAs) are small non-coding RNAs (18-22 nt) that regulate protein levels post-transcriptionally by interacting with the 3-UTR region of target mRNAs (messenger RNAs) [7]. The deregulation of miRNA expression in cancer impacts tumor biology as miRNAs regulate oncogenes and tumor-suppressor genes [8]. Moreover, miRNAs are considered excellent markers for tumor classification and prognosis [9,10]. In this context, we used the CRISPR/Cas9n system in a previous study to inactivate the oncogenic miRNA cluster *miR-17-92*, composed of six distinct miRNAs, resulting in anti-tumoral effect in an ATC cell line [11]. The sgRNAs were targeted to the upstream 3′ region of the pre-mir-17 gene that contained a binding site for the splicing factor ISY1, responsible for processing the primary transcript of miR-17-92 [12].

Since the miRNA emerged as a potent post-transcriptional regulator, seminal studies have paved the path for the investigation of miRNA deregulation in thyroid cancer [13,14,15]. Among all deregulated miRNAs, *miR-146b* is the most highly expressed miRNA in thyroid cancer [13,16,17]. Different studies have shown that *miR-146b* regulates thyroid tumorigenesis by targeting genes involved in tumor migration, invasion, and thyroid cell differentiation [18,19,20], and high levels of *miR-146b-5p* are associated with aggressive thyroid cancer [21,22].

Clinical-pathological association studies have shown that high *miR-146b* levels correlate with poor prognosis characteristics such as extrathyroidal invasion and high risk in tumor-node-metastasis staging [22]. Indeed, the detection of *miR-146b* has been tested in the blood of patients with thyroid cancer and its levels correlate with the presence of detectable disease or disease progression after surgery. Furthermore, characteristics of aggressiveness are associated with high levels of circulating *miR-146b*, such as tumor recurrence and lymph node metastasis [23,24].

Thus, *miR-146b* is a promising target to be silenced in order to study aggressive thyroid cancer biology. In this study, we show that *miR-146b* is highly expressed in ATC cell lines, and we design two sgRNAs cloned in a plasmidial CRISPR/Cas9n system to target the *MIR146B* gene in ATC. We demonstrate that gene editing of *MIR146B* with CRISPR/Cas9n is effective in disrupting the *MIR146B* gene sequence and reducing *miR-146b* expression, leading to an overall anti-tumoral effect in the ATC cell line.

## 2. Results and Discussion

### Construction of a CRISPR/Cas9n Gene Editing to Target MIR146B Gene

**Figure 1:**
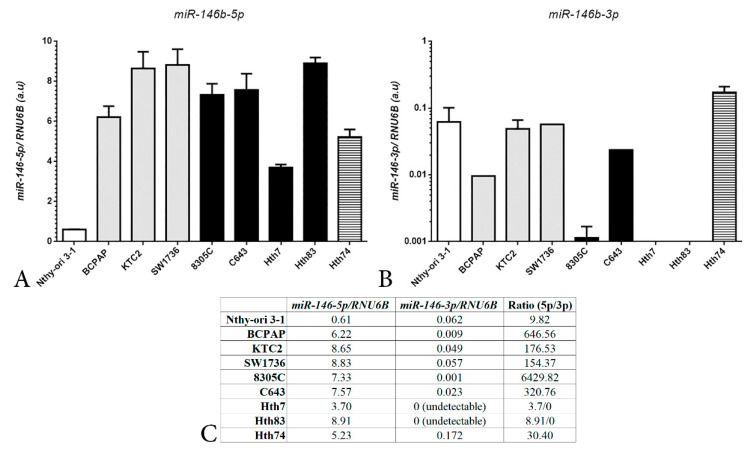
Expression levels of mature *miR-146b*-*5p* (**A**) and *miR-146b*-*3p* (**B**) in human thyroid cancer cell lines derived from PTC (BCPAP) or ATC (KTC2, 8305C, C643, Hth74 and Hth83), and as non-malignant control Nthy ori 3-1. Gene expression was calculated with Qgene using 2^−deltact^. Grey bars indicate the presence of mutation BRAF^V600E^ and black bars indicate the presence of *RAS* mutations, or *NF1* (dashed bar). (**C**) Ratio between *miR-146b-5p*/*miR-146b-3p* expression levels calculated from data shown in panel (**A**) and panel (**B**).

In order to select a cell line for *MIR146B* gene editing with CRISPR/Cas9n system, we first analyzed the expression levels of *miR-146b-5p* and *miR-146b-3p* in a panel of thyroid cancer cell lines. We observed, as expected, a high expression of *miR-146b-5p* in PTC and ATC cells (Figure 1A). It is interesting to note that the expression of the 3p strand of *miR-146b*, *miR-146b*-*3p*, is strongly reduced compared to the 5p, usually at high Ct values in qPCR (Figure 1B).

The Cancer Genome Atlas (TCGA) database shows that *miR-146b-5p* and *miR-146b-3p* are highly expressed in a fold-change comparison with controls [4]. However, our data indicate that the absolute expression of *miR-146b-5p* is 10–6000-fold higher than *miR-146b-3p*, for example in Nthy-ori 3-1 and 8305C cells, respectively (Figure 1C). Indeed, an in-depth miRNA transcriptome analysis in PTC confirms our findings, showing a high proportion of *miR-146b-5p* compared to *miR-146b-3p*, with a ratio of *miR-146b-5p/miR-146b-3p* of 44.92 [17].

Several studies have shown that the expression of *miR-146b-5p* correlates with thyroid cancer aggressiveness and the presence of the BRAF^V600E^ mutation [21,22], and also that high levels of *miR-146b* predict poorer overall survival [25]. In this context, ATC is the most aggressive and lethal histotype of thyroid cancer [26], with few successful therapeutical approaches depending on the disease progression and mutational status upon diagnosis [27]. Thus, we selected the aggressive ATC cell line KTC2 that harbors the BRAF^V600E^ mutation to validate the efficacy of CRISPR/Cas9n system.

The CRISPR/Cas9 system uses single-guide RNA (sgRNA) to direct the Cas9 endonuclease to DNA that perfectly matches the sgRNA sequence and is adjacent to the PAM sequence (NGG for Cas9 and its variants) [28]. Thus, Cas9 cleaves the double-stranded (dsDNA) and preferentially non-homologous end joining (NHEJ), DNA repair takes place, and reunites the broken DNA efficiently [2]. In this process, however, small insertions or deletions frequently occur, leading to sequence change and ultimately affecting gene expression of coding and non-coding genes.

**Figure 2:**
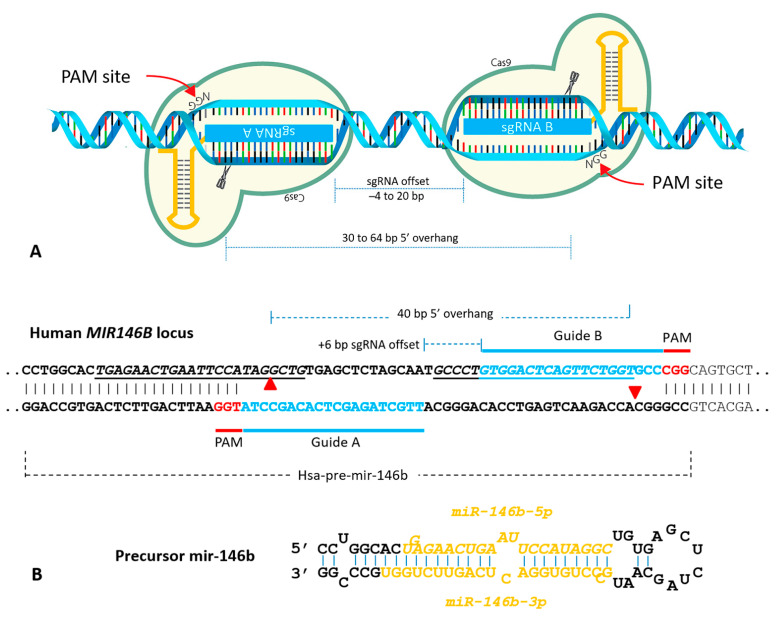
(**A**) Design of the PAM-out strategy to target human *MIR146B* with two sgRNAs in opposite strands at optimum distance. (**B**) Human *MIR146B* locus highlighting the precursor *miR-146b* sequence, and the mature strands *miR-146b*-*5p* and *3p* are underlined. SgRNA A and B target sequences are shown in blue and the PAM sequences in red. Single-strand DNA break point due to Cas9n (D10A) nickase activity is indicated by red triangle. Figure (**A**) was modified from idtdna.com to include *miR-146b* sgRNA design.

We designed a CRISPR/Cas9n plasmid system to silence *miR-146b* expression using gene editing. For that, we used the mutated Cas9n that contains the inactivating D10A mutation in the RuvC endonuclease domain of Cas9, rendering the Cas9n nickase activity (cleaves to only one strand of DNA) due to the activity of the HNH domain. Two important aspects to note for Cas9n: (1) a single-strand break can be readily repaired by ligating the single strand without changing the DNA sequence; (2) a resulting double-strand break only occurs when a concomitant single-strand break occurs at close sites in opposite strands [29]. Thus, methodologically, the CRISPR/Cas9n systems require that two sgRNAs target considerably close regions of the gene of interest and at opposite strands (optimum sgRNA offset of −4 bp to 20 bp) (Figure 2A). To this extent, we were fortunate to identify two sgRNAs with the “PAM-out” strategy to target the *MIR146B* gene, in which PAM sequences are oriented to the 3′ region of the target DNA sequence (Figure 2B). The “PAM-out” strategy has been reported to be more efficacious in generating NHEJ indels [29,30].

One advantage of using Cas9n for gene editing is the reduction of the off-targets compared to wild type Cas9, one main concern for CRISPR/Cas9 application in gene editing [29]. The individual sgRNAs used in this study, here named GuideA and GuideB (Figure 2B), have some predicted off-targets according to the ChopChop database (Supplementary Table S1). However, when using the Cas9n system, a double-strand break occurs only when both guides simultaneously target the on-target expected site, the *MIR146B* gene, minimizing off-targeted DNA break.

In order to evaluate the effect of gene editing of *MIR146B* with the CRISPR/Cas9n system, we cotransfected the KTC2 cell line with the plasmids pSpCas9n-puro-*miR-146b*-GuideA and pSpCAS9n-GFP-miR146b-GuideB, and selected the GFP-positive cells using cell sorting 48–72 h after transfection. Next, we selected puromycin-resistant cells for 7 days, generating a mixed population that was clonally separated using limiting dilution and cloning rings.

**Figure 3:**
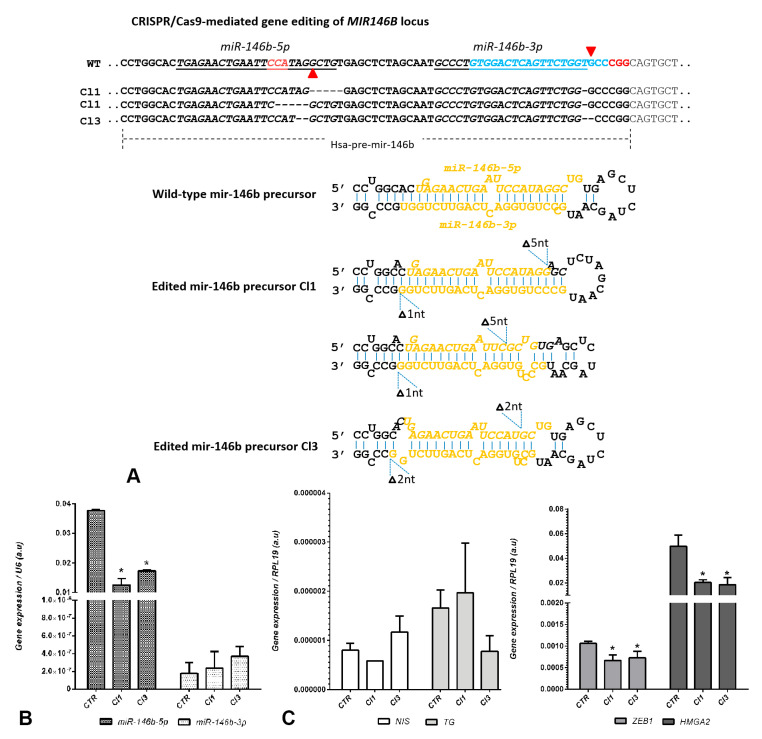
CRISPR/Cas9n-mediated gene editing of *MIR146B* gene. (**A**) Comparison of the genomic region of *MIR146B* in KTC2-Cl1 (Cl1), KTC2-Cl3 (Cl3), and KTC2-CTR (WT). Underlined sequences are the *miR-146b-5p* and *miR-146b-3p* strands of mature miRNA generated by *pre-mir-146b*. Red arrow heads indicate the points of single-strand break guided by GuideA and GuideB adjacent to PAM sequence (NGG in red). The decomposition of the sequencing traces of KTC2-Cl1 PCR shows a heterogeneous population of cells with 5 nt deletion in the *miR-146b-5p* region (either to the 3′ or 5′ of the PAM site for GuideA) and a 1 nt deletion at the *miR-146-3p* region; the KTC2-Cl3 cell line shows a homogenous population with 2 nt deletion in the *miR-146b-5p* region and a 2 nt deletion at the *miR-146b-3p* region. The prediction of precursor miRNA folding for Cl1 and Cl3 is shown as a hairpin structure compared to the control (wild-type) hairpin generated in the RNAfold WebServer program. (**B**) Expression levels of *miR-146b*-*5p* and *miR-146b-3p* after gene editing with CRISPR/Cas9n in KTC2-Cl1 and Cl3 compared to KTC2-CTR. (**C**) Gene expression of thyroid differentiation genes *NIS* and *TG*, and EMT markers *ZEB1* and *HMGA2.* Gene expression was calculated with Qgene using 2^−deltact^. * *p* < 0.05 vs. KTC2-CTR.

We derived two clonal cell lines, KTC2-Cl1 and Cl3, and the genomic DNA was validated with Sanger sequencing to confirm CRISPR/Cas9n mediated gene editing of the *MIR146B* gene (Figure 3A and Supplementary Figure S5). The analysis of sequencing traces using the SeqScreener Gene App revealed that more than 80% of sequences of KTC2-Cl1 showed a 5 nt deletion in *miR-146b-5p*, either in the 5′ or 3′ region around the GuideA cutting site (41% and 39%, respectively); while more than 93% of sequences showed a 1 nt-deletion in the *miR-146b-3p* 3′ region close to the GuideB targeted cutting site (Figure 3A, Supplementary Figures S1 and S2). For KTC2-Cl3, we observed more than 97% of sequences with a 2 nt deletion in the *miR-146b-5p* region close to the GuideA targeted PAM sequence, and more than 98% of sequences with a 2 nt-deletion in the 3′ region of *miR-146b-3p* close to the GuideB cutting site (Figure 3A, Supplementary Figures S3 and S4). These results indicate that the double sgRNA system used to target *MIR146B* is effective and produced gene editing in the desired target region. However, it is important to point out that the electropherogram of KTC2-Cl1 and Cl3 revealed the existence of traces of the wild-type sequence together with the edited ones (Figure 3A and Supplementary Figure S5), supporting the notion of monoallelic editing of *MIR146B* in both clones. Moreover, in the KTC2-Cl1 Sanger sequencing, heterogeneous sequence traces are present and indicate the existence of a heterogeneous population (Supplementary Figures S1, S2 and S5), while KTC2-Cl3 (Supplementary Figures S3–S5) shows a homogeneous clonal population.

Indeed, when we analyzed the expression of mature *miR-146b* in both clones, we observed a 60% reduction of *miR-146b*-*5p* levels in KTC2-Cl1, and 50% in KTC2-Cl3, compared to KTC2-CTR (Figure 3B). *MiR-146b*-*3p* was also detected, but at very low levels (high Ct > 37) in all cell lines. We analyzed the expression of iodine metabolizing genes such as *NIS* and *TG*, and did not detect any significant changes (Figure 3C). The Ct values for *NIS* were high (>35), indicating very low expression in KTC2 cells. We also analyzed the expression of EMT genes, such as the transcription factor *ZEB1* and *HMGA2*, and both genes showed a reduction in KTC2-Cl1 and Cl3, compared to KTC2-CTR (Figure 3C), indicating a reduction in the mesenchymal properties of KTC2 cells.

The absence of a full knock-down of *miR-146b* in KTC2-edited cells (Cl1 and Cl3) may be the result of monoallelic editing of the targeted region, but also could be linked to the addiction of thyroid cancer cells to *miR-146b* overexpression, whereas full knock-out would be lethal to cancer cells. For example, loss-of-function mutations in *miR-10b* induced lethality of the glioblastoma cells edited with CRISPR/Cas9n [31], and these mutations were only detected when screening dead floating cells. Adherent live cells showed partial knock-down of *miR-10b* (~60%), similar to our results for *miR-146b*.

Another important aspect regarding miRNA biogenesis is that the primary transcript processing to form mature miRNA strands depends on a conserved hairpin structure [32]. The microprocessor complex, composed of DROSHA and DGCR8, recognizes in the primary transcript the hairpin structure that contains: a basal junction (UG nucleotides), a lower stem (~11 nt), an upper stem (~22 nt), and the apical loop (containing UGU motif). While DGCR8 binds to the upper stem and loop, DROSHA binds to the lower stem and cuts precisely ~11 nt away from basal segment to excise the precursor (hairpin) for further processing [33]. In this context, the analysis of edited sequences of KTC2-Cl1 and Cl3 with the RNAfold program revealed alterations in the structure of the precursor of *mir-146b* (Figure 3A). KTC2-Cl1 and KTC2-Cl3 *mir-146b* precursor structures were shorter compared to KTC2-CTR, while KTC2-Cl1 also showed apical loop disruption. This perturbation in the optimal hairpin structure may impair the recruitment of the microprocessor complex and the generation of mature miRNA.

**Figure 4:**
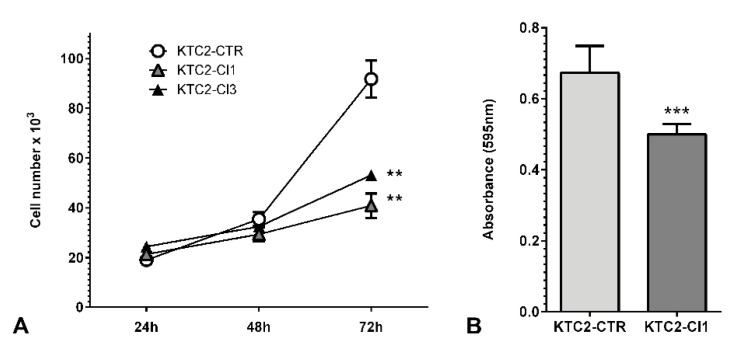
(**A**) Cell counting of KTC2-Cl1 and Cl3. Cell number was accessed at 24 h, 48 h, and 72 h after plating in comparison to KTC2-CTR. The results are representative of two independent experiments performed in triplicate. (**B**) Cell viability assay of KTC2-Cl1 and KTC2-CTR was evaluated using MTT and absorbance was measured at 595 nm. ** *p* < 0.05 vs. KTC2-CTR *** *p* < 0.01 vs. KTC2-CTR.

Next, we investigated the effect of *miR-146b* silencing using in vitro functional assays. In the process of clonal isolation from a mixed population of CRISPR-edited cells, we already observed a difference in cell growth time for KTC2-Cl1 and KTC2-Cl3 compared to KTC2-CTR. Indeed, the cell counting assay showed a significant reduction (>50%) in cell counting for KTC2-Cl1 compared to KTC2-CTR after 72 h (Figure 4A). Moreover, this effect was accompanied by a significant reduction (~25%) in cell viability of KTC2-Cl1 cells analyzed by the MTT assay (Figure 4B). For KTC2-Cl3, we observed a similar reduction in cell counting (Figure 4A).

**Figure 5:**
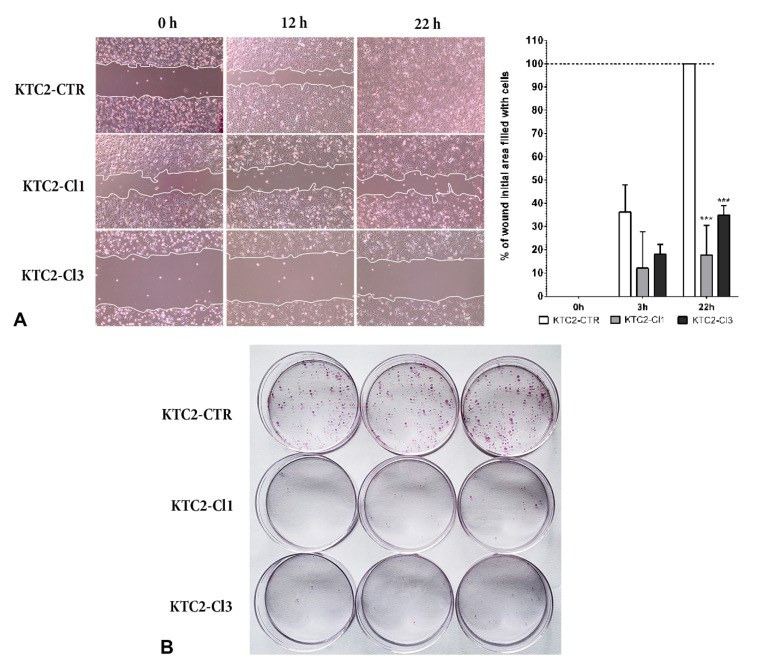
(**A**) Wound healing assay to evaluate cell migration in mitomycin treated KTC2-CTR cells compared to KTC2-Cl1 and Cl3 cells. Representative image of the gap closure soon after wound (0 h) and after 3 h and 22 h. The graph shows quantification of the area of the wound filled with cells compared to 0 h area (0% filled) using ImageJ software. *** *p* < 0.01 vs. KTC2-CTR (**B**) Colony formation assay comparing KTC2-CTR, KTC2-Cl1 and KTC2-Cl3 cells’ ability to form clones after low density seeding for 8 days. Representative results of two independent experiments performed in triplicate.

Cell migration was evaluated using a wound-healing assay while treating cells with mitomycin to block proliferation. We observed that while KTC2-CTR cells filled the wound gap in 22 h, KTC2-Cl1 and KTC2-Cl3 cells filled only ~22% and 32% of the wound area, respectively (Figure 5A). Indeed, even after 72 h, the gap in the KTC2-Cl1 and Cl3 remained open (data not shown), showing a substantial difference in cell migration.

In a previous study, our group showed that blocking *miR-146b-5p* with antimiR-LNA (antisense LNA modified RNA oligonucleotide) reduced cell migration and invasion of PTC cell lines [18]. On the other hand, the overexpression of *miR-146* in thyroid cancer cells leads to an induction of cell migration and colony formation [25]. Indeed, we also analyzed the colony formation ability of *MIR146B*-edited ATC cells, and we observed that colony formation is strongly impaired in KTC2-Cl1 and KTC2-Cl3 cells when *miR-146b* is knocked-down compared to KTC2-CTR (Figure 5B).

The colony formation assay may reflect the ability of single cells to form clones, mimicking the metastasis shedding process, and indicates that a high expression of *miR-146b* is necessary for this process. In this context, clinical-pathological association studies show that high *miR-146b*-*5p* is associated with lymph node metastasis, tumor recurrence, and a shortened overall survival [34].

**Figure 6:**
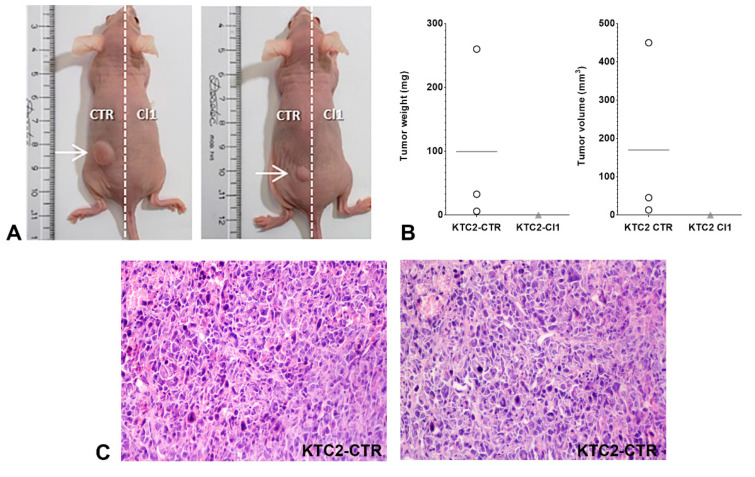
(**A**) Representative image of animals injected with KTC2-CTR cells (in the left flank) and KTC2-Cl1 cells (in the right flank). (**B**) Tumor weight and volume of KTC2-CTR cells compared to KTC2-Cl1 cells (no tumor) after animal euthanasia in the endpoint. *n* = 3 animals. ◯ KTC2-CTR; ▲ KTC2-Cl1. (**C**) Histological section of KTC2-CTR tumor stained with H&E. Magnification: 200×.

The next step was to test the potential xenograft growth of KTC2-Cl1 cells in nude mice. Our initial in vivo experiments in immunocompromised mice showed that, surprisingly, only KTC2-CTR xenotransplanted cells formed tumors in nude mice, while no tumor was detected for KTC2-Cl1 (Figure 6). The resulting KTC2-CTR tumor is a solid tumor that presents pleomorphic cells and nuclei, the presence of vascularization, and some areas of necrosis in the histological analysis (Figure 6C), which is characteristic of ATC. The strong impairment of tumor formation in KTC2-Cl1 corroborates our in vitro data that showed impaired cell counting and colony formation ability, reduction of EMT genes, and reduced cell migration in *MIR146B*-edited clones (Figure 3C, Figure 4 and Figure 5).

Our results show that *miR-146b* expression is important for cell growth, cell migration, colony formation, and tumor development, indicating the dependence of thyroid cancer cells in maintaining high levels of *miR-146b*. Moreover, recent data from liquid biopsies in thyroid cancer patients showed that circulating miRNAs have a promising role in early detection of thyroid cancer recurrence. High levels of *miR-146b* and *miR-222* are detected in recurrent thyroid cancer [23], and plasma exosomal *miR-146b*-*5p* correlates with lymph node metastasis in PTC [24].

The analysis of *miR-146b* expression in thyroid tumors shows a different degree of *miR-146b* overexpression among PTC, FTC (follicular thyroid cancer), and ATC [16]. Our data show high expression of *miR-146b*-*5p* in PTC and ATC cell lines (Figure 1A), and we developed a CRISPR/Cas9n system using two sgRNAs to target the structure of pre-mir-146 and disrupt the *miR-146b*-*5p* and *miR-146b*-*3p* proximal sequences in the genome (Figure 2). Indeed, we observed an efficient deletion of 5 nt in the *miR-146b-5p* region and a 1 nt deletion in *miR-146b-3p* region of ATC cells (KTC2-Cl1), which resulted in reduction of *miR-146b-5p* expression and antitumoral effects in vitro and in vivo.

Recent functional studies of *miR-146b* have demonstrated several new targets of *miR-146b-5p*, such as *DICER(* RNase III endonuclease that processes precursor miRNA into miRNA duplex), *PTEN*, and *RARB* [35,36,37]. Interestingly, additional studies reported a functional role for the 3p strand of *miR-146b* (*miR-146b*-*3p*) in regulating thyroid transcription factor *PAX8* and *NIS*, and the tumor suppressor *NF2* [19,38,39]. Indeed, *miR-146b-3p* inhibition restores NIS expression and radioiodine uptake in poorly differentiated thyroid cancer cells [39]. In the TCGA data from PTC, high expression of *miR-146b*-*3p* and *miR-146b-5p* was detected in BRAF-like signature tumors compared to RAS-like tumors, and correlated with thyroid differentiation status [4]. Nevertheless, our data show that *miR-146b*-*3p* has a substantial lower expression compared to the *miR-146b*-*5p* strand in PTC and ATC cell lines, irrespective of their genetic background of genetic alterations in protein-coding genes (Figure 1).

The CRISPR/Cas9 system is a promising methodology to investigate the biology of aggressive cancer cells by depleting gene sequence, correcting mutations, and some novel functions beyond gene editing [40]. For example, targeting the *TERT* gene is a promising strategy to explore its role in cancer [41], as different types of aggressive cancer show a high prevalence of *TERT* promoter mutations that over-activate *TERT* transcription [42]. In the thyroid cancer field, few initiatives have explored the complex targeting of non-coding genes with CRISPR/Cas9. In a previous study, our group successfully edited the oncogenic miRNA cluster *miR-17-92*, composed of six distinct miRNAs, with CRISPR/Cas9n [11]. For that, we targeted a conserved region involved in the recruitment of splicing factors to the primary miRNA structure [12].

In the current study, we confirm the applicability of CRISPR/Cas9n to edit a miRNA gene by demonstrating alterations in *MIR146B* gene sequence after targeting double sgRNAs and CRISPR/Cas9 nickase. As a result, the expression of *miR-146b* is reduced, leading to an overall anti-tumoral effect in the ATC cell line. This study is the first to successfully edit the *MIR146B* gene with CRISPR/Cas9n in thyroid cancer to our knowledge. We also understand that further studies with different sgRNAs and novel strategies may lead to improvement in the efficacy to target *MIR146B*, especially in the 3′ region of *miR-146b-3p.*

## 3. Materials and Methods

### 3.1. MIR146B Gene Editing Using CRISPR/Cas9n

In order to generate a DNA double-strand break with the CRISPR/Cas9n system, two sgRNAs are needed to direct the Cas9n to opposite strands of DNA, at an optimal distance of −4 to 20 bp [29], flanking the desired edition site. We used the strategy called PAM-out (Figure 2). For that, two sgRNAs that fulfil the optimal distance and the PAM-out rule in the *MIR146B* gene were designed using a combination of online available tools ChopChop and CRISPR.mit.edu [43,44]. These sgRNAs, here named as GuideA and GuideB (Figure 2B and Table 1), were cloned into the plasmids pSpCAS9n-puro (PX462), that has resistance to puromycin, and the plasmid pSpCAS9n-GFP (PX461), that encodes GFP, respectively. The GuideA cutting site is located within the 3′ region of the *miR-146b-5p* sequence, while the cutting site of GuideB is located right after the *miR-146-3p* sequence (Figure 2A), resulting in potential gene editing between both guides’ regions that may affect the precursor miRNA structure (Figure 3A), and miRNA maturation.

Briefly, the annealed oligonucleotides (Table 1) were ligated into linear plasmids digested with BbsI at a cloning site downstream of the U6 promoter, creating pSpCAS9n-puro-*miR-146b*-GuideA and pSpCAS9n-GFP-*miR146b*-GuideB. After confirming the correct insertion of the sgRNA sequence by Sanger sequencing, both plasmids were co-transfected in KTC2 cells using Lipofectamine^TM^ 2000 reagent (Invitrogen). The cells were sorted for the presence of GFP in a cytometer and treated with 1 ug/mL puromycin for 7 days. Single-cell clones were isolated by limiting dilution plating. For that, we plated ~50 cells in a 10 cm dish and, after the colonies grew, we isolated individual colonies by trypsinization using cloning rings, then replated the cells into new plates to create KTC2-CRISPR-miR146b cell line clones (that we will herein name by KTC2-Cl1 and KTC2-Cl3). We expanded these clones for the in vitro and in vivo assays. The control group was generated by the co-transfection of Empty Vectors (E.V.) of pSpCas9n-GFP and pSpCas9n-puro, with no sgRNAs cloned, creating KTC2-CTR cells.

All plasmids are from Addgene: pSpCas9n(BB)-2A-Puro (PX462) was a gift from Feng Zhang (Addgene plasmid # 48141; http://n2t.net/addgene:48141 (accessed on 20 May 2021); RRID:Addgene_48141); pSpCas9n(BB)-2A-GFP (PX461) was a gift from Feng Zhang (Addgene plasmid # 48140; http://n2t.net/addgene:48140 (accessed on 20 May 2021); RRID:Addgene_48140) [45].

### 3.2. Cell Culture

We investigated the effect of the CRISPR/Cas9n system to target *MIR146B* gene in KTC2 cells derived from human anaplastic thyroid carcinoma. Other human thyroid cancer cell lines were used for gene expression assays, and the corresponding cell culture medium are shown in Table 2. All cells were maintained in an incubator at 37 °C and 5% CO_2_. Genomic DNA was extracted from all cell lines that were authenticated using STR screening to confirm its origin as thyroid cancer.

### 3.3. Genomic DNA Sequencing for Gene Editing Validation

Genomic DNA was extracted from KTC2-CTR and KTC2-Cl1 cell lines using a DNeasy Blood and Tissue Kit (QIAGEN, Hilden, Germany). Briefly, a fragment of a *MIR146B* gene containing the CRISPR/Cas9n-targeted region was amplified in a PCR reaction using Seq Fw and Rv primers (Table 1) that amplify a 361 bp fragment. The fragment was gel-purified using a QIAquick Gel Extraction kit (QIAGEN) and sequenced using a Seq Fw primer in a Sanger sequencer. The efficiency of gene editing was evaluated with the online tool SeqScreener Gene Edit Confirmation App (SGC- https://apps.thermofisher.com/apps/gea-web/#/setup (accessed on 20 May 2021)) (Thermo Fisher Scientific, Waltham, CA, USA) by inputting the sequencing data (.ab1 file in Supplementary Material) from KTC2-CTR, KTC2-Cl1, and KTC2-Cl3, and the GuideA and GuideB sgRNA sequences.

### 3.4. Gene Expression Analysis

Total RNA was extracted using the phenol–chloroform method with the TRIzol reagent (Invitrogen-Thermo Fisher, Carlsbad, CA, USA). For miRNA expression, 10 ng of total RNA was reverse transcribed using a TaqMan^®^ Reverse Transcription Kit (Applied Biosystems, Thermo Fisher Scientific, Waltham, MA, USA) in the presence of stem-loop primers; followed by quantitative PCR using TaqMan MicroRNA Assays for *miR-146b*-*5p* (assay 1097), *miR-146b*-*3p* (assay 2361), and RNU6B (assay 1093) or U6 (assay 1973) (Applied Biosystems- Thermo Fisher Scientific, Waltham, MA, USA); and TaqMan Universal PCR Master Mix, No AmpErase^®^ UNG (Life Technologies, Thermo Fisher Scientific, Waltham, MA, USA); in a ViiA7 Sequence Detection System. MiRNA expression was normalized by comparison with RNU6B or U6 levels and calculated using the QGene program [46]. Protein coding gene expression was investigated using complementary DNA (cDNA) generated from the reverse transcription of 1 μg of total RNA using an oligo-dT primer and MMLV reverse transcriptase (Invitrogen, Thermo Fisher Scientific, Waltham, MA, USA). Quantitative PCR was performed in a ViiA7^®^ Sequence Detection System (Applied Biosystems, Thermo Fisher Scientific, Waltham, MA, USA) using SYBR Green Master Mix, cDNA, and specific primers (Supplementary Table S1). Gene expression was normalized by comparison with Rpl19 levels and calculated using the QGene program using the Ct data.

### 3.5. Cell Function Assays

#### 3.5.1. Cell Counting

Growth curves were determined using KTC2 cells by seeding 2 × 10^4^ cells per well in 12-well plates and cultivating for 24–72 h. After each of these periods, the cells were washed in PBS, removed by trypsinization, and collected. The average number of cells of the sextuplicate was determined by counting cells in a cytometer.

#### 3.5.2. Cell Viability Assay (MTT)

Cells were plated at 1 × 10^4^ cells per well in 96-well plates and cultured for 24 h. 3-(4,5-dimethylthiazol-2-yl)-2,5-diphenyltetrazolium bromide (MTT) was added to the cell culture medium to a final concentration of 250 μg/mL and incubated at 37 °C in a 5% CO_2_ incubator for 4 h. Then, the medium was removed and formazan crystals were solubilized with 0.01 M HCl in isopropanol. Absorbance was measured at 595 nm in a plate spectrophotometer SpectraMax M (Molecular Devices, San Jose, CA, USA).

#### 3.5.3. Wound Healing Assay

Cells (1 × 10^5^) were plated in 6 cm plates and cultured until confluency. Next, 2 μg/mL mitomycin (Sigma Aldrich, St. Louis, MO, USA) was added to the medium for 2 h before performing the scratch in the monolayer using a 100-μL tip. After several washes with PBS, the medium was replaced by complete medium containing 2 μg/mL mitomycin (Sigma). Wound closure was monitored until cells completely repopulated the scratch (48 h) under an inverted microscope EVOS XL Core Imaging System (Thermo Fisher Scientific, Waltham, MA, USA). Representative images were taken at several time points (from 6–48 h), and we measured the differences in the gaps and migration rate using the software ImageJ (https://imagej.nih.gov/ij/download.html, accessed on 20 May 2021).

#### 3.5.4. Colony Formation

KTC2-CTR, KTC2-Cl1, and KTC2-Cl3 cells were seeded at very low density (500 cells) in 6 cm plates and cultured for eight days. After that period, the cells were fixed with formaldehyde 3.7% and stained with 0.5% crystal violet. Representative images were captured and analyzed using ImageJ.

#### 3.5.5. In Vivo Xenotransplant in Nude Mice

For the injection in nude mice, 1 × 10^6^ cells were ressuspended in cold PBS and mixed 1:1 with Matrigel^®^ Matrix (Corning, NY, USA) in a final volume of 100 uL that was injected into opposite flanks of nude mice (left: KTC2-CTR; right: KTC2-Cl1). The evolution of tumor growth was accompanied for 12 weeks and tumor volume (V) was calculated using measurements of length (L) and width (W) of the tumor with calipers, using the formula V = (L × W^2^)/2. After euthanasia, the tumor was excised, weighted, fixed in formaldehyde, and embedded in paraffin for histological analysis. This study complied with the guidelines of the Institutional Animal Care and Use Committee (IACUC) of the Institute of Biomedical Sciences, University of Sao Paulo, registered as the protocol number CEUA n° 2023150720.

### 3.6. Statistical Analysis

The results are presented as the mean ± standard deviation, and they were submitted for analysis of variance followed by the Tukey test in GraphPad Prism 5 (GraphPad Software, San Diego, CA, USA). Differences were considered significant at *p* < 0.05.

## Figures and Tables

**Table 1:** Oligonucleotides sequence.

	Sequence 5′-3′	Experiment
GuideA Fw	**CACCG**TTGCTAGAGCTCACAGCCTATGG	cloning in PX462 plasmid
GuideA Rv	**AAAC**CCATAGGCTGTGAGCTCTAGCAA**C**	cloning in PX462 plasmid
GuideB Fw	**CACCG**GTGGACTCAGTTCTGGTGCCCGG	cloning in PX461 plasmid
GuideB Rv	**AAAC**CCGGGCACCAGAACTGAGTCCAC**C**	cloning in PX461 plasmid
*NIS* Fw	AGTACATTGTAAGCCACGATGCTGTA	qPCR
*NIS* Rv	CGGTCACTTGGTTCAGGATGA	qPCR
*TG* Fw	CCTGCTGGCTCCACCTTGTTT	qPCR
*TG* Rv	CCTTGTTCTGAGCCTCCCATCGTT	qPCR
*ZEB1* Fw	GATGACCTGCCAACAGACCA	qPCR
*ZEB1* Rv	GCCCTTCCTTTCCTGTGTCA	qPCR
*HMGA2* Fw	AAAGCAGCTCAAAAGAAAGCA	qPCR
*HMGA2* Rv	TGTTGTGGCCATTTCCTAGGT	qPCR
*RPL19* Fw	TCTCATGGAACACATCCACAA	qPCR
*RPL19* Rv	TGGTCAGCCAGGAGCTTCTT	qPCR
Seq Fw	CTGGGAACGGGAGACGATTC	sequencing of target region
Seq Rv	GAAAGCTAAGTGGAGGCCGT	sequencing of target region

**Table 2:** Cell lines and culture.

	Histology	Genetic Driver	Culture Medium
Nthy-ori 3-1	Non-malignant	none	RPMI1640 + 10% SFB + 2 mM Glutamine
BCPAP	PTC	BRAF^V600E^	DMEM + 10% SFB
KTC2	ATC	BRAF^V600E^	RPMI1640 + 5% SFB
8305C	ATC	BRAF^V600E^	RPMI1640 + 10% SFB
C643	ATC	HRAS^G13R^	RPMI1640 + 10% SFB
Hth7	ATC	NRAS^Q61R^	DMEM + 10%SFB + 2 mM Glutamine
Hth83	ATC	HRAS^Q61R^	RPMI1640 + 10% SFB
Hth74	ATC	NF1^L732F^	RPMI1640 + 10% SFB + 2 mM Glutamine

## Data Availability

The data presented in this study are available on request from the corresponding author.

## Supplementary Materials

The following are available online at https://www.mdpi.com/article/10.3390/ijms22157992/s1.
