# Supplementary material for: Targeting the Highly Expressed microRNA miR-146b with CRISPR/Cas9n Gene Editing System in Thyroid Cancer
Source: Int J Mol Sci. 2021 Jul 27;22(15):7992. doi: 10.3390/ijms22157992 (PMC8348963; doi:10.3390/ijms22157992)
Supplement: Supplementary file 1 [file ijms-22-07992-s001.zip › Supplementary Figures S1-S5 and Table S1.pdf]

Supplementary Material

Targeting the Highly Expressed microRNA miR-146b with CRISPR/Cas9n Gene Editing System in Thyroid Cancer

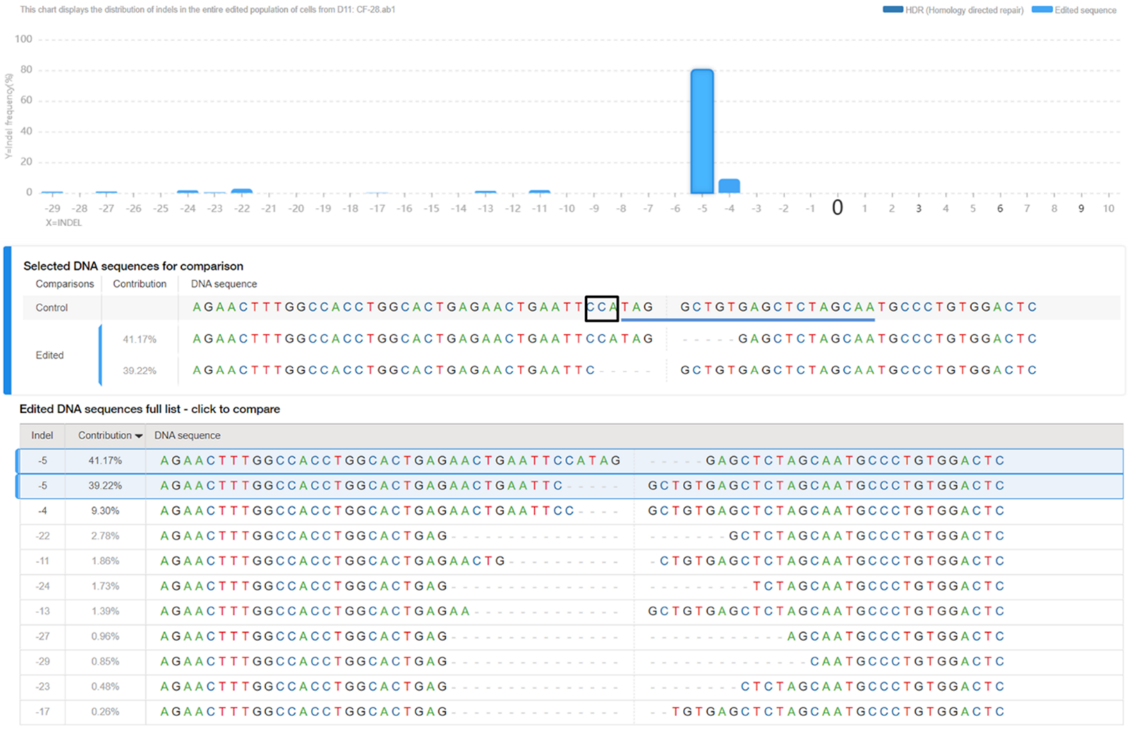

Figure S1. Validation of *MIR146B* gene editing for GuideA-targeted region in KTC2-Cl1 using the SeqScreener Gene App. Black square indicates the PAM sequence adjacent to the sgRNA underlined in blue.

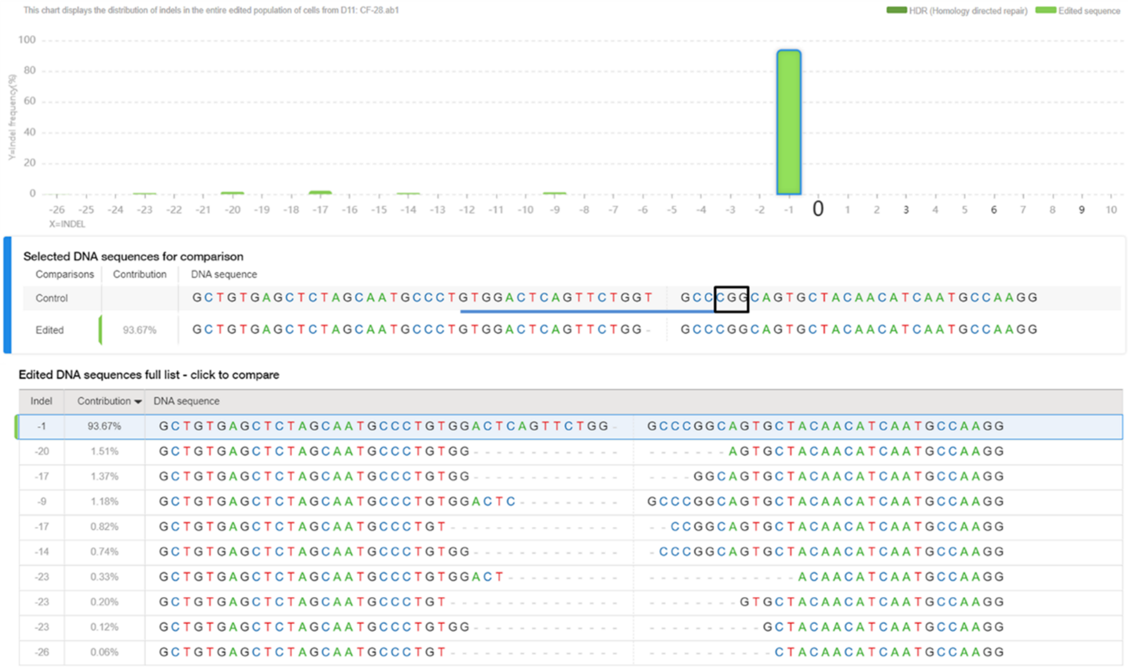

Figure S2. Validation of *MIR146B* gene editing for GuideB-targeted region in KTC2-Cl1 using the SeqScreener Gene App. Black square indicates the PAM sequence adjacent to the sgRNA underlined in blue.

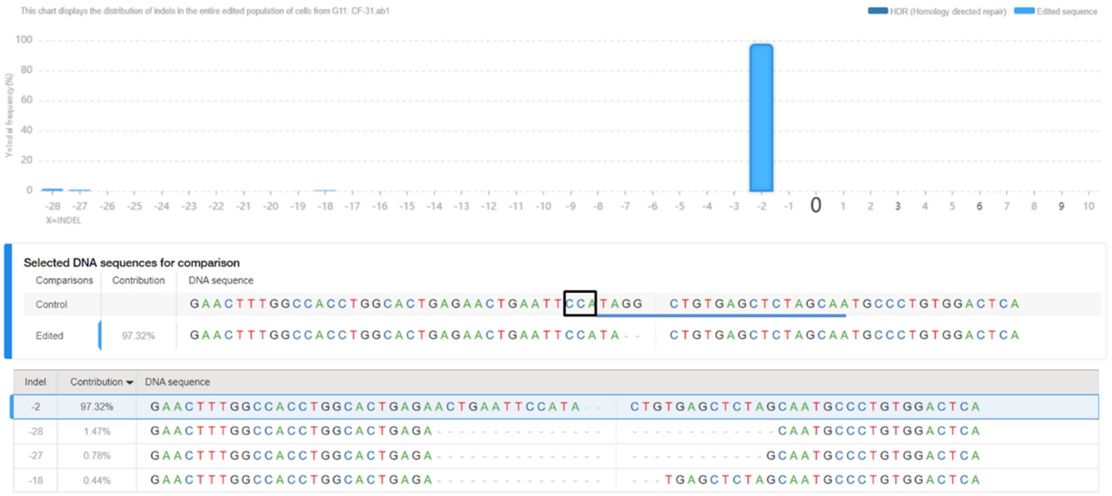

Figure S3. Validation of *MIR146B* gene editing for GuideA-targeted region in KTC2-Cl3 using the SeqScreener Gene App. Black square indicates the PAM sequence adjacent to the sgRNA underlined in blue.

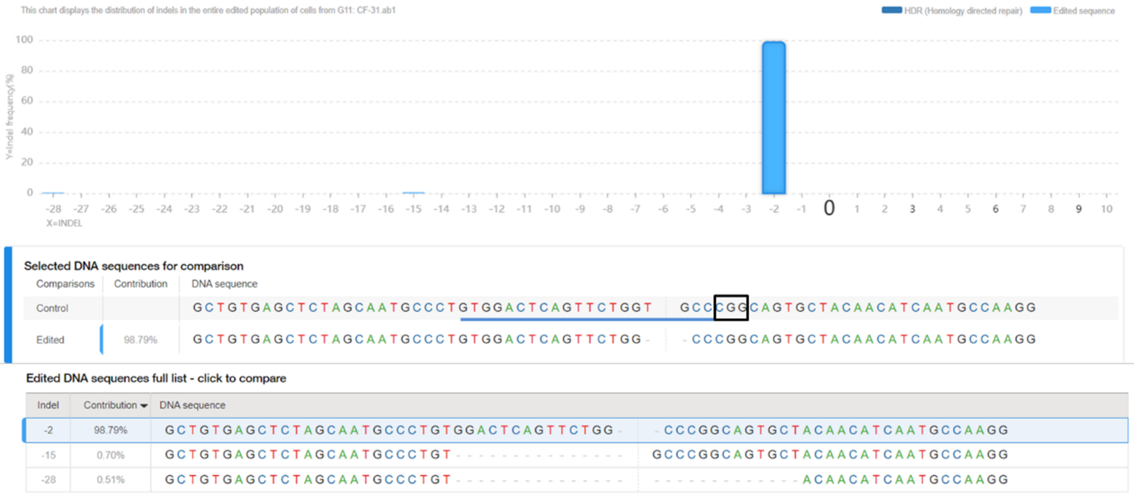

Figure S4. Validation of *MIR146B* gene editing for GuideB-targeted region in KTC2-Cl3 using the SeqScreener Gene App. Black square indicates the PAM sequence adjacent to the sgRNA underlined in blue.

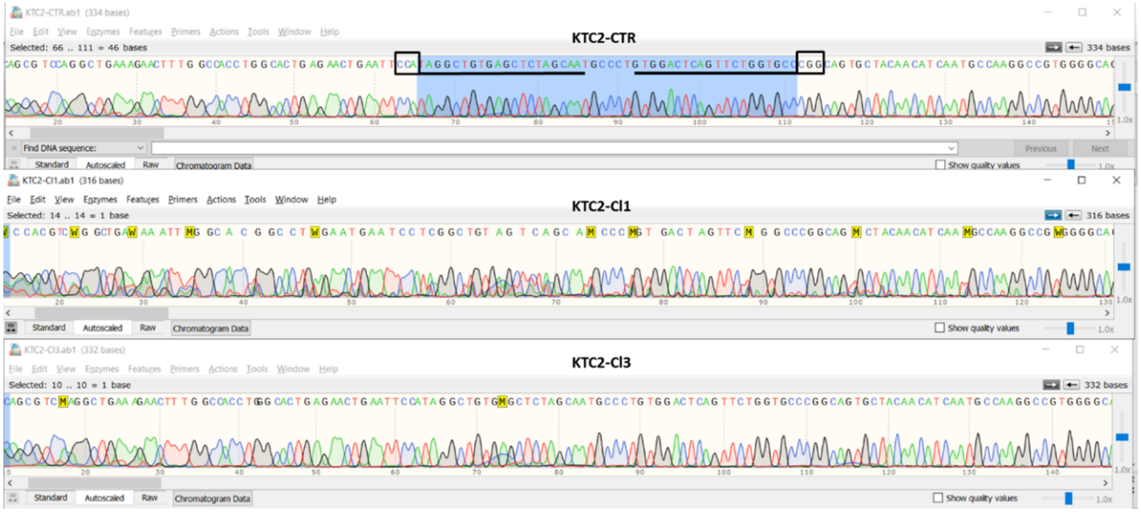

Figure S5. Raw sanger sequencing data alignment for *MIR146B* gene used in SeqScreener Gene Editing. The blue highlighted region in KTC2-CTR indicates the region between two sgRNAs that are underlined in black. PAM sequences are indicated by black squares.

Table S1. Off-targets list of sgRNA A and sgRNA B.

**sgRNA**	**Location**	**Number of Mismatches**	**Sequence (Including Mismatches)**
**sgRNA A**	chr10:102436533 (***MIR146B***) **- on target**	0	TTGCTAGAGCTCACAGCCTATGG
	chr10:95817873	3	TTGCTgtAGCTCAgAGCCTAGGG
	chr12:106688991	3	TTcCTAGAGCTCACAGtCTcAGG
	chr13:58239277	3	gTGCTAGAGCTtcCAGCCTAGGG
	chr14:98114838	3	CCCTAtcCTGTGAGCTCTAGtAA
	chr16:24336061	3	CCCTAGGCccTGAGCTCTtGCAA
	chr16:65410481	3	CCCTAGGCTGTGAaCTCTAcCAg
	chr2:221546073	3	CCTTAGaCTGTGAaCTCTtGCAA
	chr21:19900603	3	cTGCTAGAGCTtcCAGCCTAAGG
	chr5:142679117	3	TTtCTAGgGCTCACAGCtTAAGG
	chr6:122057654	2	TTGCTAGAGCTCtCAGaCTAAGG
	chr7:101906218	3	TTGCTgGAGtTCACAGCCcATGG
	chrX:139912958	3	TTGCTAGgGCTCtCAGCCTtAGG
**sgRNA B**	chr10:102436562 (***MIR146B***) **- on target**	0	GTGGACTCAGTTCTGGTGCCCGG
	chr1:22182587	3	GTGGcCTCAGTTCTGtTcCCTGG
	chr1:112028553	3	GgGGACTCAGTgCTGGTGCtAGG
	chr1:191533406	3	CCAGGCtCCAGAACTGccTCCAC
	chr13:40338408	3	CCGGGCACCAGAACTagGgCCAC
	chr14:36482672	3	CCAGGatCCAGAACTGAGTCCAt
	chr14:85131510	3	GTGGACTCAGTTaTaaTGCCAGG
	chr14:102644835	2	tTGGtCTCAGTTCTGGTGCCTGG
	chr15:30264840	3	GTaGACTCAGTgCTGtTGCCGGG
	chr15:32273795	3	CCCGGCAaCAGcACTGAGTCtAC
	chr15_KI270905v1_alt:2549795	3	GTaGACTCAGTgCTGtTGCCGGG
	chr15_KI270905v1_alt:4559480	3	CCCGGCAaCAGcACTGAGTCtAC
	chr18:23820083	3	CCTGtCcCCAGAACTGAtTCCAC
	chr19:22351022	3	CCTGGggCCAGAgCTGAGTCCAC
	chr2:127624083	3	cTGGcCTCAGTTCTGGTcCCTGG
	chr2:231859595	3	CCAGGCACCAGAgCTGAagCCAC
	chr21:45459366	3	CCGGGCACCAGAACaGAGcCCtC
	chr4:2836889	3	GTGGgCTCAGTTCTGGgGCtTGG
	chr8:144181727	3	GTGGACTCAGcTaaGGTGCCTGG
